# Pangenomic Approach To Understanding Microbial Adaptations within a Model Built Environment, the International Space Station, Relative to Human Hosts and Soil

**DOI:** 10.1128/mSystems.00281-18

**Published:** 2019-01-08

**Authors:** Ryan A. Blaustein, Alexander G. McFarland, Sarah Ben Maamar, Alberto Lopez, Sarah Castro-Wallace, Erica M. Hartmann

**Affiliations:** a Department of Civil and Environmental Engineering, Northwestern University, Evanston, Illinois, USA; b Department of Microbiology-Immunology, Northwestern University, Evanston, Illinois, USA; c Biomedical Research and Environmental Sciences Division, NASA Johnson Space Center, Houston, Texas, USA; U.S. Naval Research Laboratory

**Keywords:** International Space Station, bacterial adaptation, built environment microbiome, pangenome

## Abstract

Understanding underlying mechanisms involved in microbial persistence in the built environment (BE) is essential for strategically mitigating potential health risks. To test the hypothesis that BEs impose selective pressures resulting in characteristic adaptive responses, we performed a pangenomics meta-analysis leveraging 189 genomes (accessed from GenBank) of two epidemiologically important taxa, Bacillus cereus and Staphylococcus aureus, isolated from various origins: the International Space Station (ISS; a model BE), Earth-based BEs, soil, and humans. Our objectives were to (i) identify differences in the pangenomic composition of generalist and host-associated organisms, (ii) characterize genes and functions involved in BE-associated selection, and (iii) identify genomic signatures of ISS-derived strains of potential relevance for astronaut health. The pangenome of B. cereus was more expansive than that of S. aureus, which had a dominant core component. Genomic contents of both taxa significantly correlated with isolate origin, demonstrating an importance for biogeography and potential niche adaptations. ISS/BE-enriched functions were often involved in biosynthesis, catabolism, materials transport, metabolism, and stress response. Multiple origin-enriched functions also overlapped across taxa, suggesting conserved adaptive processes. We further characterized two mobile genetic elements with local neighborhood genes encoding biosynthesis and stress response functions that distinctively associated with B. cereus from the ISS. Although antibiotic resistance genes were present in ISS/BE isolates, they were also common in counterparts elsewhere. Overall, despite differences in microbial lifestyle, some functions appear common to remaining viable in the BE, and those functions are not typically associated with direct impacts on human health.

**IMPORTANCE** The built environment contains a variety of microorganisms, some of which pose critical human health risks (e.g., hospital-acquired infection, antibiotic resistance dissemination). We uncovered a combination of complex biological functions that may play a role in bacterial survival under the presumed selective pressures in a model built environment—the International Space Station—by using an approach to compare pangenomes of bacterial strains from two clinically relevant species (B. cereus and S. aureus) isolated from both built environments and humans. Our findings suggest that the most crucial bacterial functions involved in this potential adaptive response are specific to bacterial lifestyle and do not appear to have direct impacts on human health.

**Author Video**: An author video summary of this article is available.

## Abstract (precis)

The built environment contains a variety of microorganisms, some of which pose critical human health risks (e.g., hospital-acquired infection, antibiotic resistance dissemination). We uncovered a combination of complex biological functions that may play a role in bacterial survival under the presumed selective pressures in a model built environment—the International Space Station—by using an approach to compare pangenomes of bacterial strains from two clinically relevant species (B. cereus and S. aureus) isolated from both built environments and humans. Our findings suggest that the most crucial bacterial functions involved in this potential adaptive response are specific to bacterial lifestyle and do not appear to have direct impacts on human health.

## INTRODUCTION

Indoor surfaces and dust are widely colonized by human-associated and environmental microorganisms introduced via direct contact and passive deposition from inhabitants, transported materials, and the air supply (1, 2). Numerous metagenomics and 16S rRNA gene amplicon sequencing studies have detected diverse microbial communities on interior surfaces throughout homes, schools, offices, athletic facilities, hospitals, subway stations, and cleanrooms and aboard the International Space Station (ISS) (3–13). In addition to well-characterized bacterial survival strategies (e.g., biofilm formation and sporulation), it has been suggested that complex metabolisms, biosynthetic pathways, and antibiotic resistance genes (ARGs) may also play important roles in adaptation to these built environments (BEs) (8, 12, 14). While a variety of building features (e.g., chemical cleaning frequency, human occupancy, room type, surface materials, and ventilation) have been correlated with indoor microbial diversity (1, 2), much remains unknown about species-level population genetics associated with microbial persistence under the presumed physical and chemical selective pressures (e.g., desiccation, limited resource availability, and biocide and detergent residues from cleaning products).

The indoor microbiome has important implications for human health and safety. Elevated levels of mold (e.g., *Aspergillus*, *Cladosporium*, and *Penicillium*) are a precursor to biodegradation of building materials and can induce human development of allergies and asthma-like symptoms (15, 16). In U.S. acute-care hospitals, approximately 4% of inpatients develop nosocomial infection (closer to 10% in less industrialized countries), which leads to estimated annual economic burdens in the range of $35 to 45 billion (17, 18). Controlling the spread of hospital-acquired infections has been challenged by the widespread emergence of antibiotic-resistant pathogens, such as methicillin-resistant Staphylococcus aureus (MRSA) and vancomycin-resistant *Enterococcus* (19). Dissemination of mobile ARGs or enrichment for antibiotic-resistant organisms may be intensified by the concentration of anthropogenic chemicals from cleaning/consumer products that accumulate indoors (5, 12). Testing the hypothesis that BEs impose specific selective pressures that result in characteristic adaptive responses would advance our understanding of molecular mechanisms that could be leveraged to develop novel strategies for creating and maintaining “healthier” buildings.

Insights into potential bacterial species’ adaptability and health risks are manifested in their pangenomes, i.e., the cumulative set of genes belonging to all genomes of a taxonomic group (20, 21). Population survival under constant environmental pressures is enhanced by substantial intraspecific variation generated through rapid evolution involving mobile genetic element (MGE)-mediated horizontal gene transfer (HGT), mutation to existing genes, and DNA rearrangement or loss (22). Genomic heterogeneity within a bacterial species (or any defined taxonomic group) includes nucleotide variants within the “core” component of the pangenome (i.e., essential genes conserved across all strains) and the presence/absence of genes in the “accessory” component of the pangenome (i.e., dispensable genes in one or more, but not all, strains). Enrichments in accessory genes under specific environmental conditions may represent adaptation to the particular site or host. For example, gene presence/absence has been reported to significantly differ among *Enterococcus* isolates from human- versus environmentally sourced samples (23), as well as among *Prevotella* strains across human body sites (e.g., skin, oral cavity, and gut) (24). Moreover, the size and expansiveness of pangenomes (i.e., number of new genes discovered in each new genome analyzed) more broadly reflect a taxon’s ability to adapt and evolve (20). While relatively small and predictably bound pangenomes associate with limited lifestyles (e.g., Buchnera aphidicola, an endosymbiont of aphids), having a high propensity for increasing gene repertoire supports a more versatile metabolic and potential pathogenicity range (e.g., Bacillus cereus and Escherichia coli) (25, 26). A comparative pangenomic assessment of BE strains with human-associated and environmental counterparts would be useful to discern genetic signatures for niche-specific microbial function and biogeography.

The ISS is a relevant model system for investigating microbial adaptations to the BE due to its constant human occupancy and controlled environmental conditions (e.g., temperature, humidity, and air circulation), along with routine microbial monitoring to ensure crew safety, for nearly two decades (27). Viable members of the ISS microbiome are presumably acclimated to selective pressures of the BE (e.g., low-nutrient, dry settings) as well as spaceflight (e.g., microgravity, elevated CO_2_, and cosmic radiation). The former is underscored by ISS microbial community composition appearing more similar to that in homes on Earth than to the human microbiome (7). The hypothesis that BE conditions may have a more selective influence on microbes than spaceflight warrants investigation.

Of the several hundred bacterial strains that have been isolated from the ISS BE, B. cereus and S. aureus are among the most prevalent species in the culture collection with sequenced genomes (28–30). These economically and epidemiologically important taxa represent model organisms with drastically different lifestyles, survival strategies, and disease implications. B. cereus is ubiquitous in nature (primarily soilborne) and forms endospores (31). It is an opportunistic pathogen involved in foodborne illness (enterotoxin production) and is sometimes associated with infectious disease in immunocompromised individuals (32). In contrast, S. aureus is a highly abundant commensal within the human microbiome, often capable of biofilm formation, and increasingly implicated in nosocomial infection (e.g., MRSA) (33, 34). Accordingly, the survival dynamics of these two taxa in the ISS BE are likely distinct. While *Bacillus* spores may persist in the ISS for months or even years (35), S. aureus experiences about a 4- to 5-log reduction on surfaces over the span of a month (36). Thus, strains of the latter that have been isolated from the ISS were probably deposited from whomever was aboard during the prior few weeks, and population persistence may depend on reseeding via transfers between humans and the BE. In addition to their presence in the ISS microbiome, strains of both taxa have been frequently isolated and sequenced from various environments (e.g., BEs and soil) and human clinical samples on Earth (29–31, 37–54). In the present work, whole-genome sequencing (WGS) data from these diverse studies were leveraged to characterize the pangenomes of B. cereus and S. aureus. Our objectives were to (i) distinguish key differences in the pangenomic composition of the generalist (B. cereus) and that of the host-associated (S. aureus) model organism, (ii) determine the sets of genes and functions associated with potentially adaptive responses to the BE, and (iii) identify genomic signatures of these important members of the ISS microbiome that may present potential risk to inhabitants (e.g., ARGs, MGEs, and virulence).

## RESULTS

### Interspecies pangenome variation.

**FIG 1:**
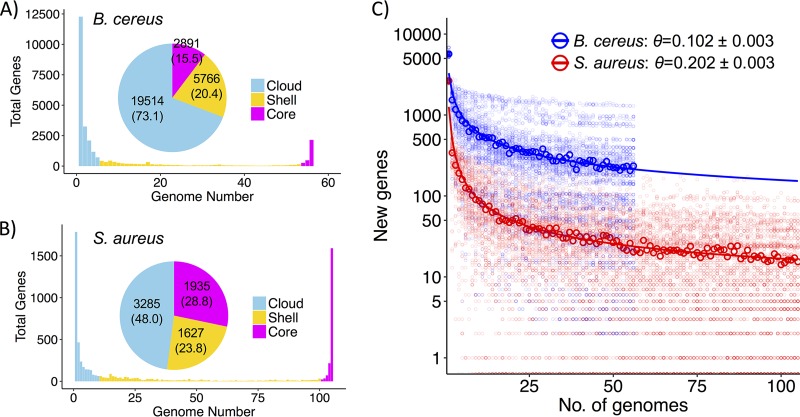
Pangenome summary statistics. (A and B) Histogram distributions of cloud, shell, and core genes. Pie chart displays numbers of total genes with percentages in parentheses. (C) Power-law fit to the mean number of new genes per genome (bold points) after 100 pangenome permutations (i.e., background points). Θ < 1 indicates that the pangenome is in the “open” state (79).

The pangenome of B. cereus contained approximately 28,171 genes, with 5,617 ± 277 genes per genome (mean ± SD) (Fig. 1; see also Fig. S1 in the supplemental material). That of S. aureus contained approximately 6,847 genes, with 2,645 ± 91 genes per genome (mean ± SD) (Fig. 1; Fig. S1). According to a power-law regression, both species pangenomes were in an “open” state (Fig. 1C). The model-predicted high likelihood of continuous discovery of new genes per genome sequenced (i.e., pangenome “openness”) indicated that populations of both species, especially B. cereus, may expand and/or alter gene repertoire over time. The predicted *N*_50_ (i.e., new genes per 50th genome analyzed) was 227.6 for B. cereus and 25.7 for S. aureus. Thus, while the two species’ average genome and pangenome sizes differed by roughly 2- and 4-fold, respectively, the numbers of new genes per genome were, disproportionately, 9 to 10 times greater for B. cereus. In summary, both pangenomes appeared boundless, though that of the generalist (B. cereus) was relatively more expansive and heterogeneous, while that of the human commensal (S. aureus) contained a more prominent core.

10.1128/mSystems.00281-18.4 FIG S1 Box plot rarefaction for total genes per genome for B. cereus (A) and S. aureus (B). Data were sourced from the total genes permutation Roary output file. Download FIG S1, TIF file, 0.6 MB. Copyright © 2019 Blaustein et al. 2019 Blaustein et al. This content is distributed under the terms of the Creative Commons Attribution 4.0 International license.

### Strain origins significantly correlate with genome contents.

**FIG 2:**
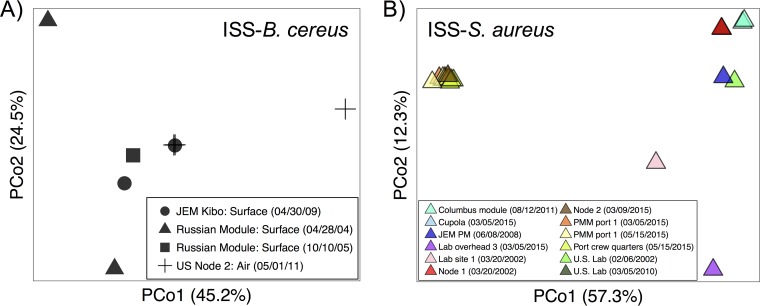
Heterogeneity in gene presence/absence among ISS-associated B. cereus (A) and S. aureus (B) strains. Symbol shape or color corresponds to sample area and date.

**FIG 3:**
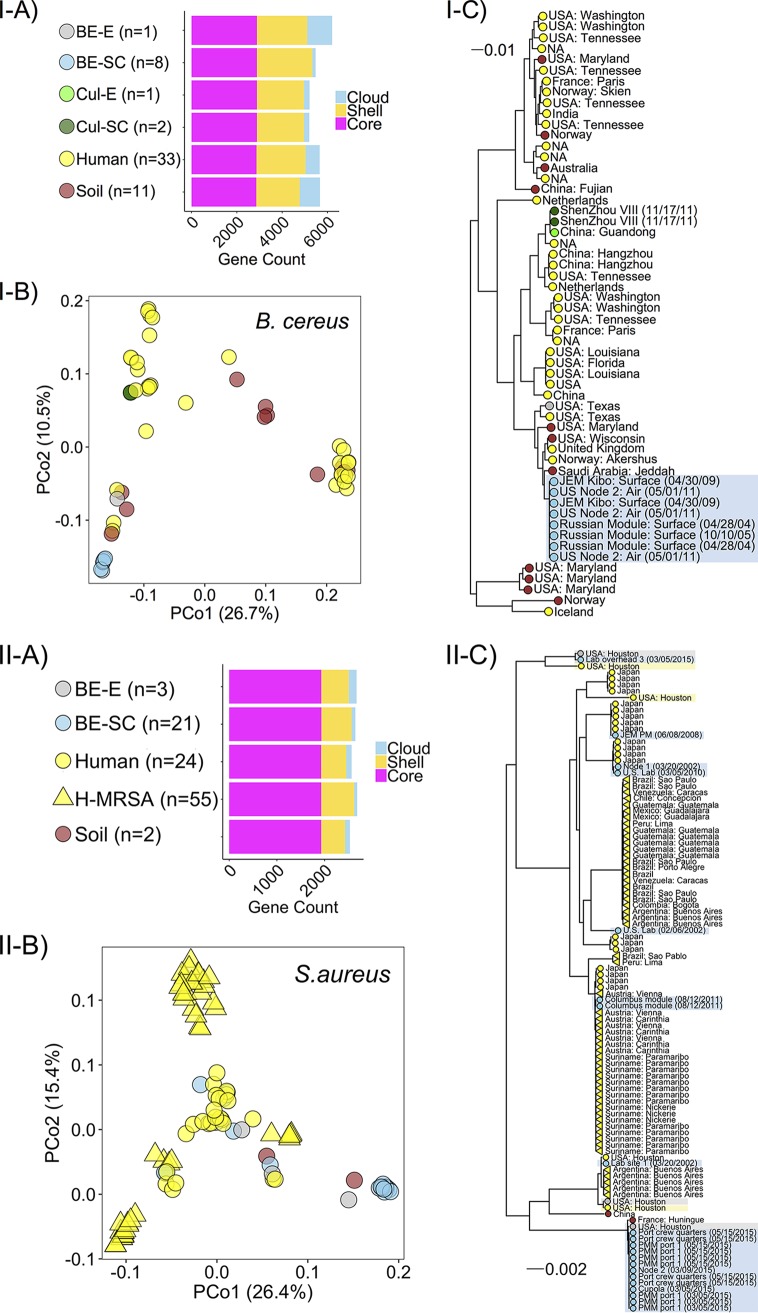
Bacterial species-level genomic diversity (i.e., gene presence/absence and core gene variants) correlates with strain origin. B. cereus and S. aureus are represented in the I and II panels, respectively. (A) Total gene counts for each fraction of the pangenome by strain origin: built environment-Earth (BE-E), -spacecraft (BE-SC), culture-Earth (Cul-E), -spacecraft (Cul-SC), human, and soil samples. BE-SC samples were taken aboard the ISS; Cul-SC samples were clonal isolates sent to space aboard the Shenzhou VIII. (B) PCoA for gene presence/absence among strains. Color/shape corresponds to sample origin. (C) Phylogenetic tree constructed from core gene codon alignment with midpoint rooting. ISS-, human-, and BE-E-sourced strains from the work of Checinska Sielaff et al. (29) and the Wallace and Voorhies data set (Table S1) are shaded in blue, yellow, and gray, respectively.

Core genome diversity significantly correlated with accessory genome diversity for both B. cereus (Mantel *r *=* *0.881, *P* = 0.001) and S. aureus (Mantel *r *=* *0.760, *P* = 0.001), suggesting that evolutionary trends for bacterial mutation have a relatively similar biogeography to gene gain/loss events. Despite large intraspecific variation in the ISS (Fig. 2), each set of strains exhibited similar genomic diversity (i.e., core gene variation and accessory gene presence/absence) in the relative context of counterpart Earth-based strains (Fig. 3B and C). Strain origin (e.g., BE-spacecraft, BE-Earth, soil, and human) significantly correlated with overall gene presence/absence for B. cereus (PERMANOVA *R*^2^ = 0.203, *P* < 0.001, *n* = 56) and S. aureus (PERMANOVA *R*^2^ = 0.233, *P* < 0.001, *n* = 105) (Table 1). Genomes of ISS-sourced isolates of each species clustered more closely with counterparts from Earth-based BEs and soil than with those from humans (Fig. 3). Importantly, genomes of the ISS-associated S. aureus were, on average, more similar to human-associated strains that were not reported as pathogens (*J *=* *0.384) than to known pathogenic variants (*J *=* *0.435), i.e., those isolated from patients with MRSA or bacteremia. These data suggest that (i) environment-based strains, regardless of being collected on Earth or in space, contain core and accessory genomic contents that are somewhat distinct from human-derived counterparts and (ii) S. aureus isolates from the ISS were more closely related to putatively commensal than pathogenic strains.

**TABLE 1:** Differences in gene presence/absence for each taxon based on strain origin, sequencing technology, sequence assembler method, culture medium, and study/reference

Taxon and factor	Variables (*n*)	*R*^2^^*a*^	*P* value^*a*^
B. cereus			
Strain origin	BE-Earth (1), BE-ISS (8), culture-Earth (1), culture-Shenzhou VIII (2), human (33), soil (11)	0.203	0.001
Culture medium^*b*^	B. cereus selective agar (8), brain heart infusion medium (2), custom medium (6), fastidious broth to blood agar (3), HEPA filter to R2A agar (3), Luria-Bertani medium (8), Trypticase soy agar (3)	0.327	0.002
Sequencing technology	Illumina (32), 454 (12), combination (10)	0.100	0.002
Assembler	A5 (3), ABySS (10), CANU (1), Celera (4), CLC NGS Cell (2), IDBA-UD (5), combination (5), Newbler (11), SOAPdenovo (3), SPAdes (8), Velvet (1)	0.323	0.001
Study	19 different studies/NCBI references (Table S1)	0.484	0.001
S. aureus			
Strain origin	BE-Earth (3), BE-ISS (21), human (24), human-MRSA (55), soil (2)	0.233	0.001
Culture medium^*b*^	Brain heart infusion agar (2), HCH-supplemented liquid medium (1), Trypticase soy agar (28), Trypticase soy broth (37)	0.218	0.001
Sequencing technology	Illumina (32), PacBio (15)	0.027	0.004
Assembler	A5 (13), CLC Genomics Workbench (51), PacBio HGAP3 (15), SeqMan NGen (16), SOAPdenovo (1), SPAdes (8), Velvet (1)	0.385	0.001
Study	9 different studies/NCBI references (Table S1)	0.472	0.001

a PERMANOVA results for genome associations with each factor.

b Reflects the medium that was used in initial bacterial isolation or that which was used in isolate collection/processing, where available (i.e., not all studies provided culture method details, and some provided only information for how strains were processed rather than initial isolation).

10.1128/mSystems.00281-18.8 TABLE S1 Accession numbers, metadata, and CheckM assembly statistics for genomes utilized in the pangenomic meta-analysis. Download Table S1, XLSX file, 0.1 MB. Copyright © 2019 Blaustein et al. 2019 Blaustein et al. This content is distributed under the terms of the Creative Commons Attribution 4.0 International license.

To discern genetic signatures that may be associated with spaceflight conditions (e.g., microgravity and radiation) or from BE conditions (e.g., desiccation and chemical cleaning product residues), we focused on genomes from a study where clonal B. cereus isolates were sent to space aboard the Shenzhou VIII in containers where they were grown in Luria-Bertani medium (49). It was reported that after 16 days in spaceflight, compared to Earth-based controls that were cultivated the same way, the strains developed three polymorphic loci and experienced changes in growth rate, antibiotic resistance, and levels of metabolic expression and function (49). Despite these mutations and physiological changes that occurred in response to spaceflight conditions, the strains that were sent to space aboard the Shenzhou VIII in culture medium did not become more similar to the set of spacecraft BE strains (i.e., in Fig. 3, part I, Cul-SC does not diverge from Cul-E, and both remain distinct from BE-SC). This may be a reflection of the Cul-SC/E samples growing in a rich medium for a relatively short time, while the BE-SC samples were likely not growing for some time prior to sampling. We can still infer that spaceflight alone was probably not responsible for the drastic genomic profile differences in the ISS versus counterpart strains (Fig. 3); BE conditions may have played a role as well, with an influence from sampling date and location (i.e., ISS interior site) (Fig. 2).

To evaluate correlations between genome content and strain origin without potential biases associated with study-specific factors (e.g., sampling location and date, criteria used to select strain for further cultivation and sequencing, and factors displayed in Table 1), we focused on the subset of genomes from the Wallace and Voorhies data set (Table S1). S. aureus had been isolated from the ISS-BE (*n* = 8), preflight BE (*n* = 3; cargo bags and hardware surfaces), and preflight astronauts (*n* = 4; human skin swabs). Pairwise PERMANOVA indicated no differences between gene presence/absence in preflight BE and the ISS-BE isolates (*R*^2^ = 0.134, *P* = 0.177) or in preflight BE and preflight astronaut isolates (*R*^2^ = 0.089, *P* = 0.972). Alternatively, there were subtle, yet not significant, differences in gene presence/absence between preflight astronaut and ISS-BE isolates (*R*^2^ = 0.139, *P* = 0.097). While the BE surface strains may resemble “local” human-associated commensal strains, potential genomic differences in strains from the ISS-BE and humans may reflect site-specific factors.

### Function enrichments in isolates from the ISS, soil, and humans.

Inferring potential microbial adaptive responses to a particular environment requires focusing on genome-encoded functions. A total of 2,907 and 1,729 unique functions (not counting “hypothetical proteins”) were encoded in B. cereus and S. aureus pangenomes, respectively. Consistent with the trends for gene presence/absence and core gene variation, there were significant differences in bacterial functional profiles based on strain origin (B. cereus PERMANOVA *R*^2^ = 0.209, *P* < 0.001, *n* = 56; S. aureus PERMANOVA *R*^2^ = 0.299, *P* < 0.001, *n* = 105) (Fig. S2).

10.1128/mSystems.00281-18.5 FIG S2 Bacterial species-level genomic diversity based on gene product (encoded function) presence-absence correlates with sample origin: built environment-Earth (BE-E), -spacecraft (BE-SC), culture-Earth (Cul-E), -spacecraft (Cul-SC), human, and soil. All BE-SC samples were taken aboard the ISS, and the culture samples were clonal isolates sent to space aboard the Shenzhou VIII. Download FIG S2, TIF file, 1.2 MB. Copyright © 2019 Blaustein et al. 2019 Blaustein et al. This content is distributed under the terms of the Creative Commons Attribution 4.0 International license.

**FIG 4:**
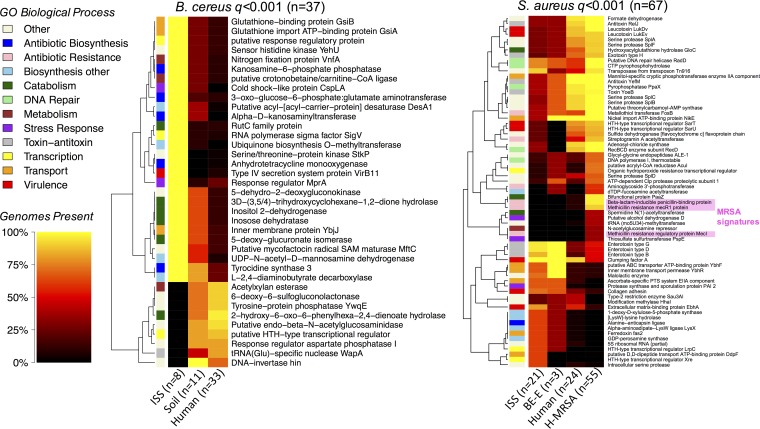
Strain origin-enriched gene products. The heat map displays all function enrichments with FDR *q *<* *0.001 for B. cereus (left) and S. aureus (right). Heat color corresponds to percentage of genomes per origin type containing at least 1 gene encoding the listed product. Row colors indicate the biological process group for gene products. On the S. aureus panel, the functions associated with the staphylococcal cassette chromosome *mec* (IWG-SCC 2009) are shaded.

According to the generalized linear model (GLM), 262 B. cereus functions and 104 S. aureus functions were significantly associated with strain origin (*P* < 0.01 and FDR *q *<* *0.1) (Table S2). The most strongly associated functional enrichments (FDR *q *<* *0.001) are displayed in Fig. 4. For both taxa, greater proportions of ISS and Earth-based BE strains than human strains encoded key functions involved in material transport, antibiotic biosynthesis (i.e., kanosamine, tetracycline, and tyrocidine in B. cereus; bacilysin in S. aureus), and other biosynthetic processes (i.e., fatty acids and ubiquinone in B. cereus; amino acids, isoprene, and lipopolysaccharides in S. aureus) (Fig. 4; Table S2). ISS-associated B. cereus strains were also enriched with unique metabolism (i.e., carbohydrate and nitrogen), catabolism (i.e., aromatic hydrocarbon and inositol), and stress response (cold shock and starvation) processes (Fig. 4; Table S2).

10.1128/mSystems.00281-18.9 TABLE S2 Bacterial gene product correlations with strain origin. Gene products with significant correlation (*P* < 0.01 and FDR *q *<* *0.1) are shaded, and those with highest significance (*q *<* *0.001) are in bold. For the latter, the UniProtKB pathway, gene ontology (GO) molecular function, and GO biological process are listed (i.e., manual search at http://www.uniprot.org; visited on 14 June 2018). The designated GO biological process (i.e., antibiotic biosynthesis, antibiotic resistance, biosynthesis other, catabolism, DNA repair, metabolism, ribosome, stress response, toxin-antitoxin, transcription, transport, virulence, or other) was selected in accordance with the cumulative UniProtKB information. Download Table S2, XLSX file, 0.3 MB. Copyright © 2019 Blaustein et al. 2019 Blaustein et al. This content is distributed under the terms of the Creative Commons Attribution 4.0 International license.

Regarding implications for astronaut health, there were several virulence factors (i.e., the UniProtKB biological process was “virulence” or “pathogenesis”) enriched in the ISS-derived B. cereus (i.e., type IV secretion system protein Vir11B) and S. aureus (i.e., clumping factor A, collagen adhesion, and extracellular matrix-binding protein EbhA) (Fig. 4). Additionally, several resistance functions were enriched in the genomes of ISS-derived strains compared to Earth-based counterparts (i.e., multidrug efflux pumps for B. cereus; beta-lactamase and heavy metal for S. aureus), though to a lesser extent (i.e., 0.01 < *q* < 0.1) than the above lifestyle-associated processes with *q *<* *0.001 (e.g., biosynthesis, catabolism, material transport, and metabolism), as these resistances were often common in isolates from humans/soil elsewhere (Tables 2 and 3).

**TABLE 2:** Antibiotic resistance gene products enriched by strain origin (*P* < 0.01 and FDR *q *<* *0.1) for *B. cereus* for origins with *n* ≥ 3^*a*^

B. cereus ARG product	Antibiotic class or other	% of strains from origin (*n*):			FDR *q*
		BE-ISS (8)	Soil (11)	Human (33)	
Metallothiol transferase FosB 2	Fosfomycin	0.0	45.5	84.8	0.001
UDP-4-amino-4-deoxy-l-arabinose–oxoglutarate aminotransferase	Polymyxin	0.0	54.5	0.0	0.005
**Multidrug resistance protein EbrB**	**Multidrug**	**100.0**	**27.3**	**57.6**	**0.045**
Beta-lactamase 3	Penicillin	0.0	63.6	18.2	0.077
Cadmium resistance transcription regulatory protein CadC	Heavy metal*	0.0	9.1	24.2	0.077
**Multidrug resistance protein Stp**	**Multidrug**	**100.0**	**63.6**	**100.0**	**0.080**

a Percentage of strains from the respective origins encoding each function are listed. Gene products that were more frequently present in ISS strains than soil- and/or putatively commensal human-derived strains are displayed in bold. *, not an antibiotic class, though can be associated with antibiotic resistance.

**TABLE 3:** Antibiotic resistance gene products enriched by strain origin (*P* < 0.01 and FDR *q *<* *0.1) for S. aureus for origins with *n* ≥ 3^*a*^

S. aureus ARG product	Antibiotic class	% of strains from origin (*n*):				FDR *q*
		BE-ISS (21)	BE-Earth (3)	Human (24)	Human-MRSA (55)	
Beta-lactam-inducible penicillin-binding protein	Penicillin	0.0	0.0	8.3	96.4	<0.001
Methicillin resistance MecR1 protein	Penicillin	0.0	0.0	8.3	90.9	<0.001
Aminoglycoside 3′-phosphotransferase	Aminoglycoside	0.0	33.3	0.0	58.2	<0.001
Metallothiol transferase FosB	Fosfomycin	38.1	66.7	62.5	100.0	<0.001
Streptogramin A acetyltransferase	Streptogramin	23.8	0.0	50.0	87.3	<0.001
Methicillin resistance regulatory protein MecI	Penicillin	0.0	0.0	0.0	34.5	<0.001
Bleomycin resistance protein	Antitumor	0.0	0.0	4.2	30.9	0.006
**Cadmium resistance transcription regulatory protein**	Heavy metal*	**95.2**	**66.7**	**54.2**	**92.7**	**0.007**
Kanamycin nucleotidyltransferase	Aminoglycoside	0.0	0.0	8.3	30.9	0.017
Macrolide export ATP-binding/permease protein MacB	Macrolide	4.8	0.0	29.1	43.6	0.031
**Beta-lactamase**	**Penicillin**	**100.0**	**100.0**	**70.8**	**96.4**	**0.036**

a Percentage of strains from the respective origins encoding each function are listed. Gene products that were more frequently present in ISS strains than soil- and/or putatively commensal human-derived strains are displayed in bold. *, not an antibiotic class, though can be associated with antibiotic resistance.

Although gene product presence/absence for S. aureus isolated from the ISS BE was correlated with year of sampling (PERMANOVA *R*^2^ = 0.692, *P* < 0.001, *n* = 21) and study/reference (PERMANOVA *R*^2^ = 0.467, *P* < 0.001, *n* = 21), several gene products enriched (or absent) in the ISS-BE were generally conserved (Fig. S3). None of the BE genomes contained MRSA signatures (i.e., beta-lactam-inducible penicillin-binding protein, MecR1 methicillin resistance protein, and methicillin resistance regulatory protein MecI) that were, conversely, present in human-associated strains. Human-associated pathogenic S. aureus happened to also be enriched with additional antibiotic resistances (e.g., macrolide, fosfomycin, and streptogramin), virulence factors, and DNA repair processes (Fig. 4; Table 3). Similarly, compared to the ISS strains of B. cereus, those that were soil- and/or human-borne encoded additional resistances more frequently (i.e., fosfomycin, polymyxin, penicillin, and heavy metal) (Table 2). Collectively, these data suggest that microbial adaptations to the ISS/BE are largely related to general lifestyle responses involving biosynthesis, material transport, metabolism, and stress tolerance. As these enriched gene products are part of broader functional pathways, and KEGG pathways encoded in the genomes that we leveraged appeared to correlate with phylogeny as measured by core gene distance (not strain origin *per se*, at least for S. aureus) (Fig. S4), it remains somewhat unclear whether the BE selects for overall functional potential of bacteria.

10.1128/mSystems.00281-18.6 FIG S3 Profile of S. aureus strain origin-enriched gene products (*q *<* *0.001; *n* = 67) for the ISS isolates (*n* = 21). Heat map displays gene product presence (yellow)/absence (black) for the gene products from Fig. 4. Reference and sampling date correspond to colors shown in the top two rows. Download FIG S3, TIF file, 1.7 MB. Copyright © 2019 Blaustein et al. 2019 Blaustein et al. This content is distributed under the terms of the Creative Commons Attribution 4.0 International license.

10.1128/mSystems.00281-18.7 FIG S4 (A) Phylogenetic tree constructed from core gene codon alignment with midpoint rooting (same as in Fig. 3, part II), from which one BE-SC isolate from each branch containing a BE-SC isolate and one counterpart isolate on the same branch were processed for KEGG pathway analysis in GhostKOALA (M. Kanehisa, Y. Sato, and K. Morishima, J Mol Biol 428:726–731, 2016, https://doi.org/10.1016/j.jmb.2015.11.006). Note that only 53.3% ± 1.3% of the open reading frames were able to be annotated with KEGG Ontology (mean ± SD; *n* = 14). (B) PCoA illustrates significant association between KEGG profile (collapsed to “C” level) and tree branch position (PERMANOVA *R*^2^ = 0.633; *P = *0.002). Download FIG S4, TIFF file, 1.7 MB. Copyright © 2019 Blaustein et al. 2019 Blaustein et al. This content is distributed under the terms of the Creative Commons Attribution 4.0 International license.

**FIG 5:**
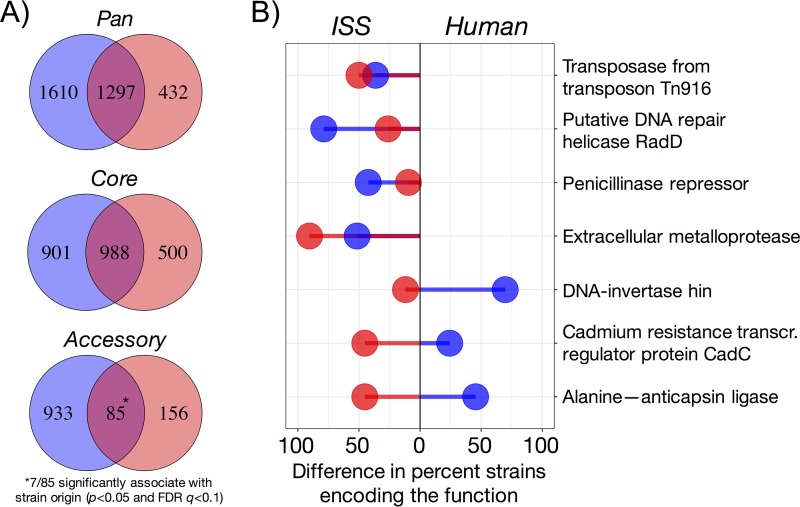
Overlap in gene products across taxa. (A) Numbers of shared and distinct functions encoded in the pangenomes, core genomes, and accessory genomes of B. cereus (blue) and S. aureus (red). (B) Shared accessory gene products across taxa that significantly correlated (*P* < 0.01 and FDR *q *<* *0.1) with strain origin for origins containing *n* ≥ 3 strains (i.e., B. cereus: ISS, human, soil; S. aureus: ISS, BE-Earth, human, human-MRSA). Each segment corresponds to the differences in percentage of strains (blue, B. cereus; red, S. aureus) isolated from the ISS-BE and human samples (i.e., putatively commensal S. aureus only, not MRSA) encoding the gene product. For example, functions with both segments in the same direction demonstrate association with the same origin or vice versa for segments in the opposite direction.

Gene products conserved across taxa enriched in a distinct environment may reflect more fundamental bacterial adaptations to said environment. Since B. cereus and S. aureus are both Gram-positive members of the same phylum, *Firmicutes*, we anticipated an overlap in most of their core and some of their accessory functions. Indeed, 52.3% of B. cereus core gene products were also core in S. aureus, and 66.4% of S. aureus core gene products were also core in B. cereus (Fig. 5A). Of the 85 overlapping accessory functions, 7 were significantly associated with strain origin (*P* < 0.01 and FDR *q *<* *0.1). Focusing on ISS-BE and putatively commensal human-associated strains only, 4 of these functions were encoded more frequently in the former for both taxa, while the 3 others were more frequently encoded in human-associated B. cereus and ISS-associated S. aureus compared to respective counterparts (Fig. 5B). For example, an MGE-associated transposase and penicillin resistance regulatory protein were more common in the ISS strains of both taxa (Fig. 5B), suggesting a potential implication for interspecies ARG mobilization in the confined environment. In contrast, bacilysin biosynthesis (i.e., involving alanine-anticapsin ligase) and a cadmium resistance regulator were more often associated with ISS-derived S. aureus and human-derived B. cereus (Fig. 5B). Perhaps the functions with opposing sample origin associations may play a role in bacterial persistence away from traditional niches (i.e., human-derived S. aureus in the BE and environment-borne B. cereus in humans).

### Potentially mobile functions unique to the ISS.

To identify potentially mobile functions that correlated with the ISS, we characterized the genes that associated with enriched MGEs. In an effort to limit potential sequencing technology and assembler method biases (Table 1), we limited the scope of this analysis to the B. cereus genomes from Illumina-based studies with raw reads available from NCBI-SRA (*n* = 22) and used a standardized sequence assembly method (i.e., all SRA files were processed with SPAdes whereas the original assemblies had been processed with ABYSS, Celera, IDBA-UD, CLC NGS Cell, or combinational approaches, as described in Table S1). There were 18/22 genome assemblies that passed quality control, which was similar to the proportion for the original assemblies that were processed (Table S1). Notably, there was significantly less variation in gene presence/absence of annotated genomes for the new assemblies (*J *=* *0.338 ± 0.014; mean ± SE) compared to the original assemblies (*J *=* *0.362 ± 0.013) (Wilcoxon *P* = 0.048). This finding further supports the concept of sequence assembler bias (55) and suggests that comparative genomics studies should use a standardized approach, if possible.

Scoary (56) analysis of the pangenome constructed from the new B. cereus assemblies identified significant correlations between ISS strains and *pepF1* (product: oligopeptidase F, plasmid) (*P* = 0.001, FDR *q *<* *0.021), *bin3* (product: putative transposon Tn552 DNA-invertase bin3) (*P* < 0.001, FDR *q *=* *0.004), and *Int-Tn* (product: transposase from transposon Tn*916*) (*P* = 0.001, FDR *q *=* *0.021) (Table S3). Because *bin3* and *Int-Tn* were each only present in 3 human strains and 1 soil strain, while all non-ISS strains also encoded a variant of *pepF1* (Table S3), we focused on the two former for subsequent analysis.

10.1128/mSystems.00281-18.10 TABLE S3 Scoary output displaying full list of genes significantly associated with ISS-BE B. cereus genomes for the subset of B. cereus strains with raw sequencing data from Illumina-based studies processed with our standardized pipeline (*n* = 22). We note that this analysis was not performed for S. aureus due to limited raw sequencing data available (*n* = 1 as shown in Table S1). Download Table S3, XLSX file, 0.1 MB. Copyright © 2019 Blaustein et al. 2019 Blaustein et al. This content is distributed under the terms of the Creative Commons Attribution 4.0 International license.

Local neighborhood genes (i.e., flanking ±5 genes on the contig) of the two transposon-associated genes were characterized to test the hypothesis that the ISS strain-specific MGEs may (i) potentially carry different genes on the same MGE in counterpart strains and (ii) present potential risk for astronaut health (i.e., carry transmissible ARGs or virulence factors). *Int-Tn* was linked with *lysN* (product: 2-aminoadipate transaminase), *ddl* (product: d-Ala–d-Ala ligase), and *tenA* (product: aminopyrimidine aminohydrolase) in 75%, 63%, and 63% of the ISS strains, respectively (Table S4). That is, the transposase in ISS isolates consistently associated with lysine, thiamine, and peptidoglycan biosynthesis. In contrast, *Int-Tn* in human- and soil-derived strains appeared to associate with different genes altogether. In addition, *bin3* was found to associate with *rapG* (product: response regulator aspartate phosphatase G) in 87.5% of ISS strains, but none of the Earth-based counterparts (Table S4). Considering the variability in B. cereus sampling time and location within the ISS and the local intraspecific genome variation (i.e., Japanese module on 30 April 2009, Russian module on 28 April 2004 and 10 October 2005, and U.S. node on 1 May 2011, as indicated in Fig. 2), these two MGEs and the mechanisms they may mobilize (i.e., biosynthesis and stress response pathways) are likely important for persistence in the ISS. Moreover, there was no indication that the ISS-enriched MGEs carried ARGs, which supports the hypothesis that the ARGs in the ISS-borne B. cereus and S. aureus were intrinsic and not acquired after deposition to the BE.

10.1128/mSystems.00281-18.11 TABLE S4 Local neighborhood genes of *bin3* and *Int-Tn* in B. cereus. The genes (and gene products, in parentheses) that are located 1 to 5 positions upstream or downstream from each copy of the putative MGE are displayed, along with contig number and base pair range. “HP” indicates hypothetical protein, and “NA: end contig” indicates that the location at the upstream or downstream position is nonexistent due to reaching the end of the contig. Download Table S4, XLSX file, 0.05 MB. Copyright © 2019 Blaustein et al. 2019 Blaustein et al. This content is distributed under the terms of the Creative Commons Attribution 4.0 International license.

## DISCUSSION

We present the first study to use comparative pangenomics to uncover underlying genes and functions that may be involved in microbial colonization and persistence in the BE. The pangenomes of two common members of the indoor microbiome with high economic importance, B. cereus and S. aureus, were defined from WGS data of strains isolated from the ISS (i.e., a model BE), BEs on Earth, soil, and humans. Consistent with prior studies on these taxa, the numbers of new genes per genome indicated substantial intraspecific variation and overall pangenome “openness” associated with a broad niche range (25, 26, 33, 57). Indeed, B. cereus and S. aureus are versatile in host- and environmentally associated microbiomes as commensal or pathogenic variants (31, 44). Comparing the two pangenomes indicated that the host-associated taxon contains a more dominant fraction of core genes, while that of the generalist is more boundless, perhaps to a disproportionate extent (i.e., ratios of new genes per genome were exceedingly greater than ratios in average genome size and total pangenome size). This disparity may partially reflect genomic biases associated with different culture methods for the two taxa, since media and even preparation technique influence which strains will grow (58). Nevertheless, the associations we observed for both B. cereus and S. aureus genome contents with strain origins, along with the key distinctions in their pangenomes and general lifestyles, highlight the potential for characteristic microbial response to the BE.

Our findings suggest that diverse biological processes play a central role in bacterial adaptation to the BE; i.e., genetically distinct members of introduced populations may endure the local selective pressures (e.g., desiccation, limited resource availability, and biocide and detergent residues from cleaning products) (12, 59). Strains of B. cereus and S. aureus isolated from the ISS and Earth-based BEs (i.e., B. cereus from Earth-based BEs unable to be compared due to low sample size; *n* = 1) were enriched with functions involved in biosynthesis (e.g., fatty acids, amino acids, and antibiotics), catabolism (e.g., sugars and aromatics), material transport, metabolism (e.g., nitrogen and carbohydrate), and stress response (e.g., cold shock), compared to their respective human- and/or soil-derived counterparts. We recognize that correlations identified between strain origin and genomic content may have been influenced, at least partially, by potential biases associated with isolate genomic content across studies due to study-specific factors: e.g., methods used for isolate collection randomization, “batch effects” associated with sample processing, sampling date and location, and sequencing technology and assembler, etc. As such, the relative genomic similarity in BE strains and heterogeneity among Earth strains may reflect the scarcity of BE WGS data available (i.e., to our knowledge, we leveraged all available WGS data for B. cereus and S. aureus isolated from the BE, which are largely limited to the ISS). When evaluating correlations in genomic content and strain origin without such biases (i.e., focusing on the subset of S. aureus genomes from the Wallace and Voorhies data set; Table S1), we still found subtle differences in overall gene presence/absence between strains isolated from the ISS-BE and preflight astronauts. Of course, these differences may reflect site-specific factors (i.e., different humans were the source of S. aureus in the ISS). To confirm the hypothesis of a bacterial adaptive response to presumed selective pressures in the BE, there is an urgent need for future studies designed to control for the above limitations and expand culture/WGS data repositories for BE strains. Longitudinal sampling of clinically relevant isolates collected from the BE and human occupants in parallel (e.g., ISS or on Earth, such as in a hospital setting), across several locations, warrants investigation.

Genes that confer antibiotic resistance (e.g., β-lactamases, heavy metal resistance, and multidrug efflux) may play a significant role in BE selection as well (8, 12, 60). In fact, long-term microbial exposures to benzalkonium chloride, the primary cleaning disinfectant used on interior surfaces of the ISS (13, 61), are known to influence ARG dissemination (62) and could possibly select for intrinsic mutations that confer resistance (e.g., *mdep* expression, decreased porin uptake, and changes in cell wall composition) (63). However, in our analysis, the role of ARGs in ISS selection was less emphasized than that suggested from metagenomics assessments in other BEs (e.g., hospitals and athletic facilities) (8, 12). The lack of associations between BE strain origins and a larger number of ARGs may be due to (i) discrepancies between culture-independent and -dependent analyses (e.g., biases toward specific, cultivable organisms depending on the culture medium used), (ii) reduced transmission of undesirable strains (e.g., multidrug-resistant pathogens) to the ISS BE because of preflight health monitoring/screening (64), (iii) potential limitations to uncovering the absolute ARG diversity (e.g., gene annotation sensitivity yielding uncharacterized hypothetical proteins), or (iv) the spaceflight environment not necessarily selecting for the same ARGs as BEs on Earth. Perhaps Earth-based BEs may demonstrate more selection for ARGs than the ISS due to human occupancy-dependent microbial transfers and long-term evolution. While hospital staff members work at the same facility for years at a time, ISS astronauts are cycled in and out every few months. The strong correlation we found between S. aureus overall gene product presence/absence and sampling date in the ISS, along with the inactivation rates of this taxon (36), suggests that the BE isolates were probably deposited from whomever was aboard during the prior few weeks. The role of microbial reseeding and cycling between surface and host in propagation of ARGs within a population is an interesting avenue for future research.

Microbial selection in the ISS may have been influenced by selective pressures from spaceflight (e.g., microgravity, elevated CO_2_, and radiation) and/or BE conditions (e.g., desiccation, limited resource availability, and biocide and detergent residues from cleaning products). Importantly, physiological responses of bacteria in the BE, specifically the ISS, were partially consistent with genomic signatures we identified. The phenotypes of B. cereus were reported to be nonvirulent (i.e., non-toxin-producing and lacking toxin-encoding plasmids pXO1 and pXO2) (30). Our genomic assessment further indicated an absence of *cytK* and *nhe*, which encode other toxins commonly associated with B. cereus pathogenicity (38). Additionally, spaceflight analog culture investigations have demonstrated that S. aureus adopts a colonization phenotype with a repression of virulence characteristics (65). Culture-based resistance assays had also previously indicated that the majority of both sets of strains were resistant to penicillin and some S. aureus strains were resistant to erythromycin and rifampin as well (29, 30). In the present work, we found that only penicillin resistance was significantly enriched in the ISS-associated S. aureus genomes, which may simply reflect the fact that ARGs identified in the ISS genomes were either not conserved or sometimes common in counterpart strains elsewhere. Moreover, spaceflight conditions alone (i.e., separate from BE) undoubtedly influence genomic and physiological responses, despite being potentially less evident than adaptations to the BE. Spaceflight and microgravity simulations have been reported to enhance growth, virulence, biofilm formation, nutrient scavenging, stress tolerance, and/or antimicrobial resistance of B. cereus, Cupriavidus metallidurans, Escherichia coli, Micrococcus luteus, Pseudomonas aeruginosa, *Salmonella* sp., and S. aureus *in vitro* (49, 65–73). In the present study, not uncovering enrichments related to these functions (aside from stress tolerance) suggests that microbes in the ISS, and even in other BEs, may undergo potential physiological changes that are not necessarily reflected in their genomes as gene gains or losses. It may also likely reflect the low-humidity, well-ventilated environment of the ISS not being conducive to bacterial growth; i.e., bacteria in the BE do not need nutrients *per se* but only to withstand stresses associated with being stranded on surfaces or in dust. To reconcile differences in microbial physiological and genomic responses to the ISS and distinguish selective effects of spaceflight from BEs, time-series *in situ* experiments on genomic, transcriptomic, and proteomic dynamics of microbial isolates and communities on surfaces warrant investigation.

While mobile genetic elements (MGEs; e.g., plasmid, transposon, and phage) that mediate horizontal gene transfer (HGT) may enhance bacterial population survival under constant environmental pressures, they are also responsible for the dissemination of genes involved in antimicrobial resistance and virulence (74). In this study, we identified two transposon genes (*Int*-*Tn* and *bin3*) frequently associated with the same genes encoding putatively beneficial biological processes (i.e., biosynthesis and stress tolerance) in the ISS B. cereus genomes. Since acquired mobile genes are representative of the unique history of the microorganism, uncovering these similarities further supports the role of these functions in potential adaptation to the ISS BE. Biofilm formation/incorporation, which creates opportunities for gene exchange between bacteria (75), may be enhanced during spaceflight (68). However, it remains unclear whether the HGT involving *Int*-*Tn* and *bin3* actually occurred in the BE, as this was probably unlikely. Barriers to HGT on surfaces/in dust may include (i) physical distance separating microbes, (ii) lack of moisture sources that may otherwise enable mobility and nutrient transport, (iii) general stresses that induce dormancy, and/or (iv) lack of compatibility between strains. Thus, an alternative explanation is that similar strains/spores have persisted in the closed system for long durations of time. Since B. cereus is a sporeformer, it is possible that the isolates were a result of bacteria that were deposited months to years before sampling or were from dust brought aboard with supplies. Regardless, our findings suggest that the few ARGs that were enriched in the ISS strains were likely intrinsic and not mobile/acquired. Testing the hypothesis that interactions in BE microbiota may mediate enhanced bacterial survival and, potentially, virulence and resistance dissemination is an interesting area for future research.

Overall, our comprehensive pangenomic analysis suggests that members of the BE microbiome, both on Earth and in the ISS, contain characteristic genomic signatures distinct from human- and/or soil-derived counterpart strains. Such signatures involve complex biological processes that may reflect local adaptations, the most crucial of which do not appear to have direct impacts on human health.

## MATERIALS AND METHODS

### Genome assembly processing.

GenBank genome assemblies for 83 strains of B. cereus (76 B. cereus and 7 *Bacillus* sp., grouped with B. cereus in this text) and 106 strains of S. aureus that were isolated from spacecraft, humans, or soil were retrieved from the NCBI Assembly Database. Accession numbers and associated metadata (e.g., strain origin, location, culture medium, sequencing technology, and assembler) are listed in Table S1 in the supplemental material. Assembly quality was evaluated with CheckM v1.0.7 (76), and genomes with less than 97% completeness or greater than 3% contamination were excluded from further analyses. The remaining genomes (B. cereus, *n* = 56; S. aureus, *n* = 105) were annotated with Prokka v1.12, referencing the respective genus (77). Output .gff files were processed in Roary v3.12.0 with minimum blastp identity of 90% to build pangenome matrices (78).

### Pangenome analyses.

Statistical analyses and data visualization were performed in R v3.2.1. Genes were grouped into categories of “cloud,” “shell,” and “core” corresponding to presence in <10%, 10 to 95%, and >95% of genomes analyzed, respectively. Power-law regression was used to estimate the size and expansiveness of each pangenome based on 100 random permutations of new genes per genome: *N*(*n*) = α · *n*^−θ^, where *N* is the expected number of genes, *n* is the number of genomes sequentially added, and θ determines whether the pangenome is open (<1) or closed (>1) (79).

The associations between the presence/absence of genes and strain origin, culture medium, sequencing technology, sequence assembler, and study were evaluated with principal coordinate analysis (PCoA) and permutational analysis of variance (PERMANOVA) using Jaccard’s index with binary standardization as the beta diversity metric. To infer phylogenetic similarity, the core gene amino acid sequence alignments that were generated from Roary were processed with FastTree v2.1.10 using the Jones-Taylor-Thornton model and CAT approximation (80). The Newick trees were processed with Phangorn v2.4.0 (81) for midpoint rooting and plotted with Ape v5.1 (82). Correlation between evolutionary diversity within each taxon (i.e., core genome distance) and diversity in accessory genome content was evaluated with the Mantel test. We further assessed phylogenetic similarity associations with functional diversity via a PCoA and PERMANOVA for potential correlation between core gene alignment tree branch position and KEGG pathway gene ontology abundances, as determined with GhostKOALA (83), for select BE- and human-associated strains.

In search of microbial functions enriched by strain origin, we evaluated gene product presence/absence associations with strain origin (for origins with *n* ≥ 3) using a generalized linear model (GLM) with binomial error distribution. The resulting *P* values were adjusted to *q* values using the Benjamini-Hochberg false discovery rate (FDR) procedure (84), and associations with *P* < 0.01 and *q *<* *0.1 were considered significant. The list of gene products with significant strain origin enrichments was screened for those that may confer antibiotic resistance via manual search for appropriate keywords (e.g., “resistance,” “lactamase,” “macrolide,” and “tetracycline”). This keyword-based approach is supported by the notion that Roary groups genes based on percent identity and assigns each group a gene/gene product name based on the most common annotation. For positive hits in the screen, UniProtKB (http://www.uniprot.org) was used to confirm antibiotic resistance as the biological process (e.g., ensure that it was not antibiotic biosynthesis).

### ISS MGEs: controlling for batch effect biases.

A subset of the genome assemblies had raw sequence data available in the NCBI Sequence Read Archive (*n* = 35/189; see Table S1 in the supplemental material). To remove potential biases associated with sequencing and assembly protocol (Table 1), the raw sequence data for B. cereus strains from the paired-end Illumina sequencing studies (*n* = 22/83 B. cereus genomes) were downloaded through the SRA toolkit v.2.8.1 and processed with a standardized pipeline. We note that this analysis was not able to be performed for S. aureus due to limited raw sequencing data available (*n* = 1; Table S1). For the 22 B. cereus genomes, Trim Galore v0.4.4 (85) was utilized to remove residual adapter sequences and trim reads at nucleotides with a Phred score below 30. Genomes were assembled *de novo* with SPAdes v3.12.0 using default parameters (86). Scaffolds that passed CheckM quality assessment (*n* = 18/22 scaffolds; note, that since only 56/83 original assemblies had passed quality control, a limited number of raw sequence data sets yielding high-quality genomes was expected) were further processed for functional annotation and pangenome matrix construction using methods described above. The Wilcoxon test was applied to assess differences in gene presence/absence variation (i.e., Jaccard distance) in genomes annotated from the standardized assemblies compared to the original assemblies.

Scoary v1.6.16 was used to identify genes significantly associated with the ISS strains compared to all other sample types (56). MGE-related gene products (e.g., transposons) that were significantly associated with sample type (*P* < 0.01 and FDR *q *<* *0.1) were further analyzed for similarities in local neighborhood genes (i.e., ±5 flanking genes before or after the MGE on the contig).

All data and bioinformatics and R scripts that may be used to reproduce our analyses are available at https://github.com/hartmann-lab/BE_ISS_pangenomes.
