# Supplementary figures and images for: Pangenomic Approach To Understanding Microbial Adaptations within a Model Built Environment, the International Space Station, Relative to Human Hosts and Soil
Source: mSystems. 2019 Jan 8;4(1):e00281-18. doi: 10.1128/mSystems.00281-18 (PMC6325168; doi:10.1128/mSystems.00281-18)

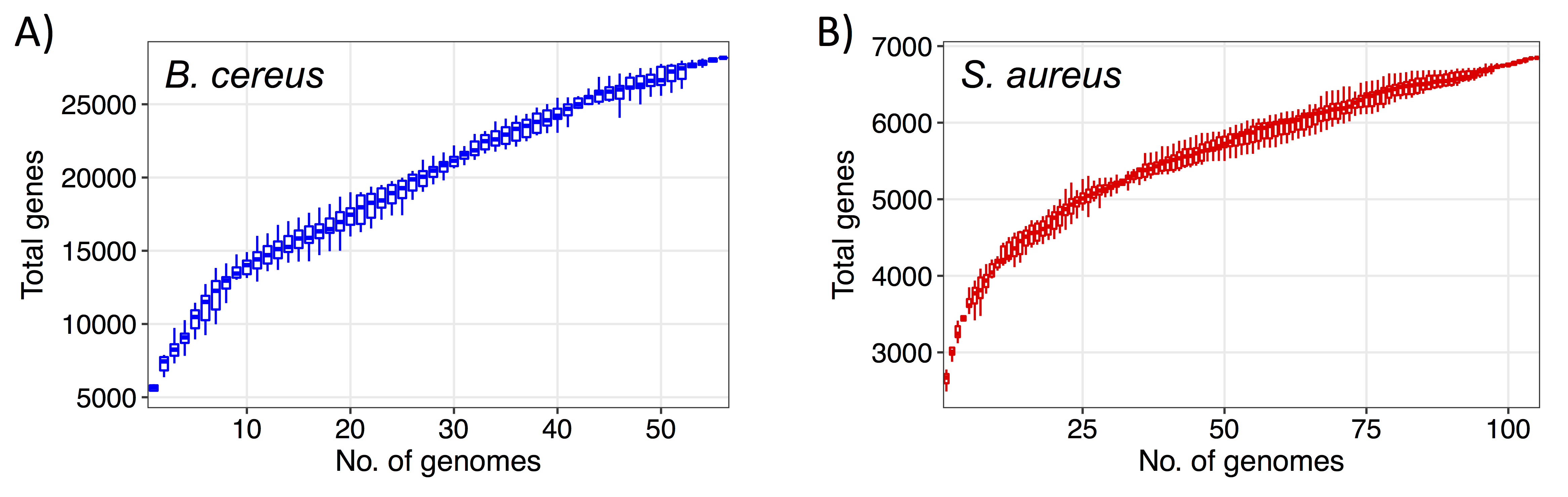

Supplement: FIG S1 [file sys001192310sf1.tif]

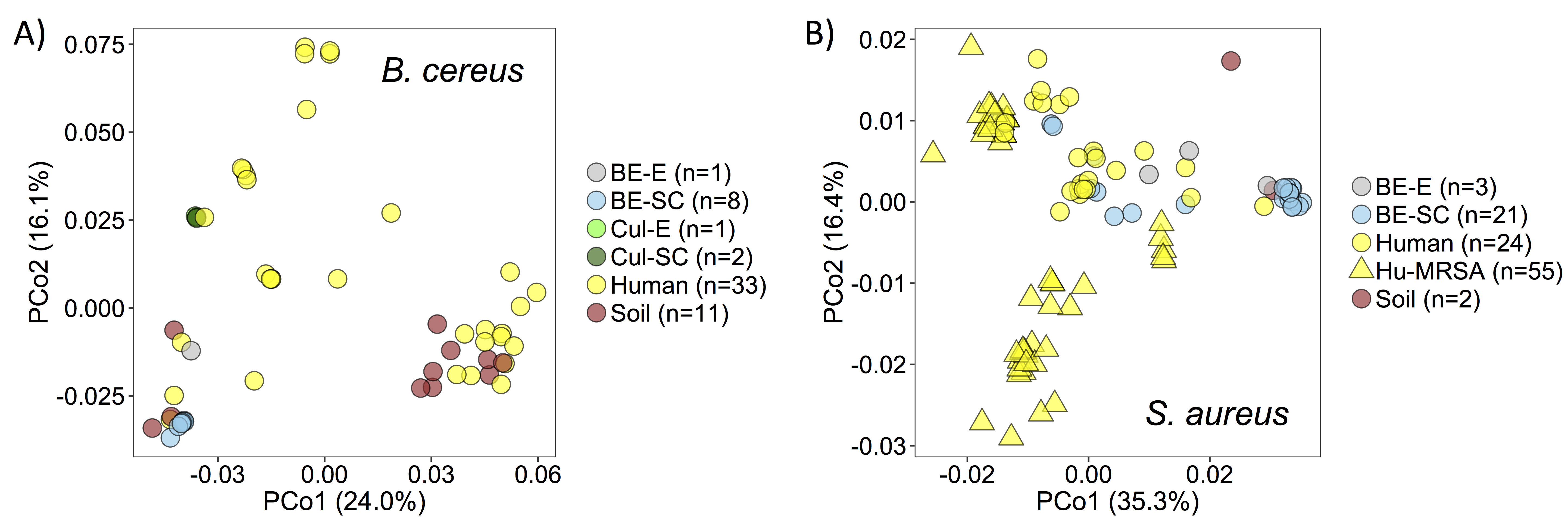

Supplement: FIG S2 [file sys001192310sf2.tif]

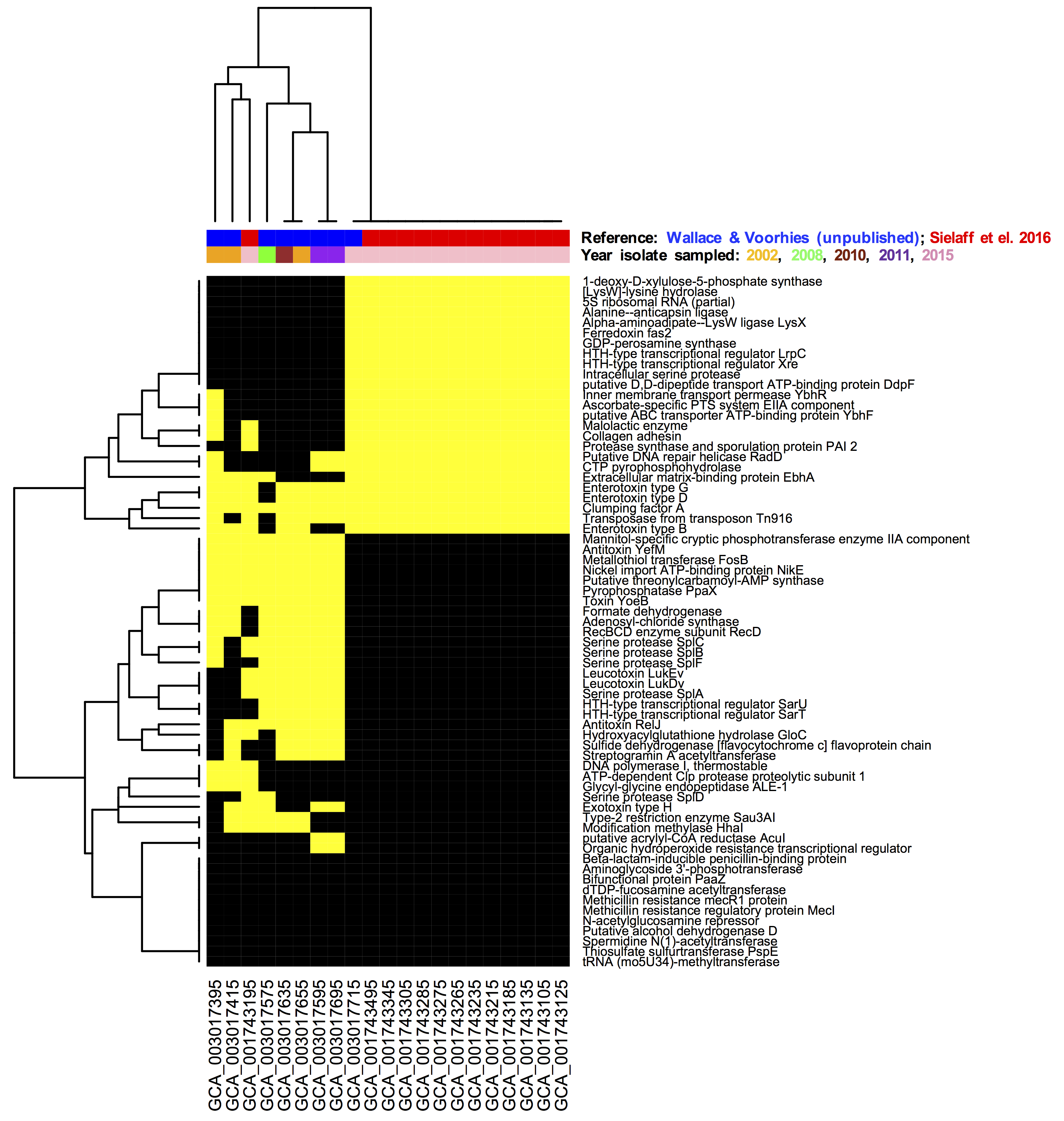

Supplement: FIG S3 [file sys001192310sf3.tif]

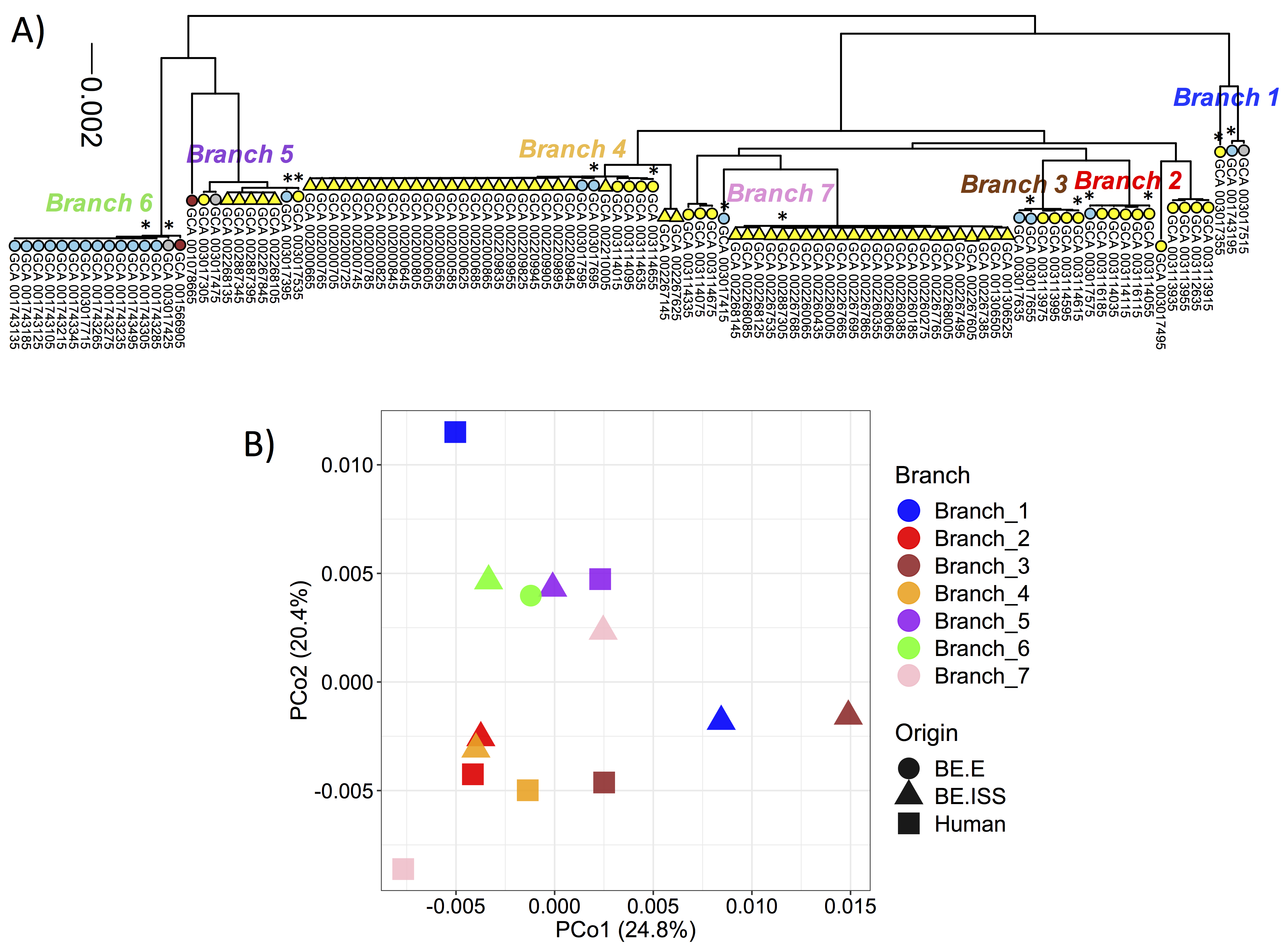

Supplement: FIG S4 [file sys001192310sf4.tiff]
